# Dying child and nurses’ mourning

**DOI:** 10.1192/j.eurpsy.2021.1075

**Published:** 2021-08-13

**Authors:** A. Zartaloudi, C. Lekas, I. Koutelekos, E. Evangelou, E. Kyritsi

**Affiliations:** 1 Nursing, University of West Attica, Athens, Greece; 2 Intensive Care Unit, Henry Dunant Hospital, Athens, Greece

**Keywords:** Nurse, care, death, Child

## Abstract

**Introduction:**

One of the most complex and emotional aspects of nursing is the interaction between the nurse and the dying child. The attitudes of nurses towards death, affect the quality of care.

**Objectives:**

To investigate pediatric nurses’ attitudes towards death.

**Methods:**

Methodology: 170 nurses, working in pediatric hospital departments completed a questionnaire which included sociodemographic characteristics and information related to their previous training and clinical experience regarding death issues in general and dying children’s care in particular.

**Results:**

68.6% reported that the death of a child affects them very much, while 44.7% of the participants didn’t feel well prepared to manage death issues. Pediatric nurses were greatly affected by children’s death, expressing mainly feelings of sadness (44%), compassion (22%), guilt (22%) and anger (22%). 73% of the sample wished the hospitalized child, died when they were not present. 53.5% had been trained regarding the care of dying patients and the management of death and mourning as part of their curriculum and 21.2% had attended a relative seminar / lecture. The importance of proper and adequate education becomes particularly apparent considering that the majority of our sample either did not feel sufficiently prepared in order to deal with death and mourning, even though more than 70% of our participants had been relatively educated.

**Conclusions:**

The incorporation of the notions of death and care at end of life in the theoretical and practical fields of nursing will improve the quality of services offered at the end of life for patients and their families.

